# P-2330. Herpes Zoster in Mexican Patients With Cancer. Clinical Features, Complications and Economic Burden

**DOI:** 10.1093/ofid/ofae631.2482

**Published:** 2025-01-29

**Authors:** Pamela Alatorre Fernandez, Erick Antonio Osorio López, Maura González Guerrero, Antonio Camiro Zúñiga, Beda Islas-Muñoz, Patricia Volkow-Fernández, Patricia Cornejo Juarez

**Affiliations:** Instituto Nacional de Cancerología, Mexico City, Distrito Federal, Mexico; None, Mexico City, Distrito Federal, Mexico; Instituto Nacional de Cancerología, Mexico City, Distrito Federal, Mexico; Instituto Nacional de Cancerología, Mexico City, Distrito Federal, Mexico; Instituto Nacional de Cancerologia, México, Distrito Federal, Mexico; INSTITUTO NACIONAL DE CANCEROLOGÍA, MEXICO CITY, Distrito Federal, Mexico; Instituto Nacional de Cancerología, Mexico City, Distrito Federal, Mexico

## Abstract

**Background:**

Herpes zoster (HZ) typically causes a painful, unilateral, vesicular rash that affects one dermatome. In immunocompromised, it can disseminate and cause postherpetic neuralgia. The incidence of HZ has increased in recent years, especially in elderly and immunocompromised people, cancer patients included. Mexico has a low vaccination rate with no policy for patients at high risk for HZ.

**Figure:**
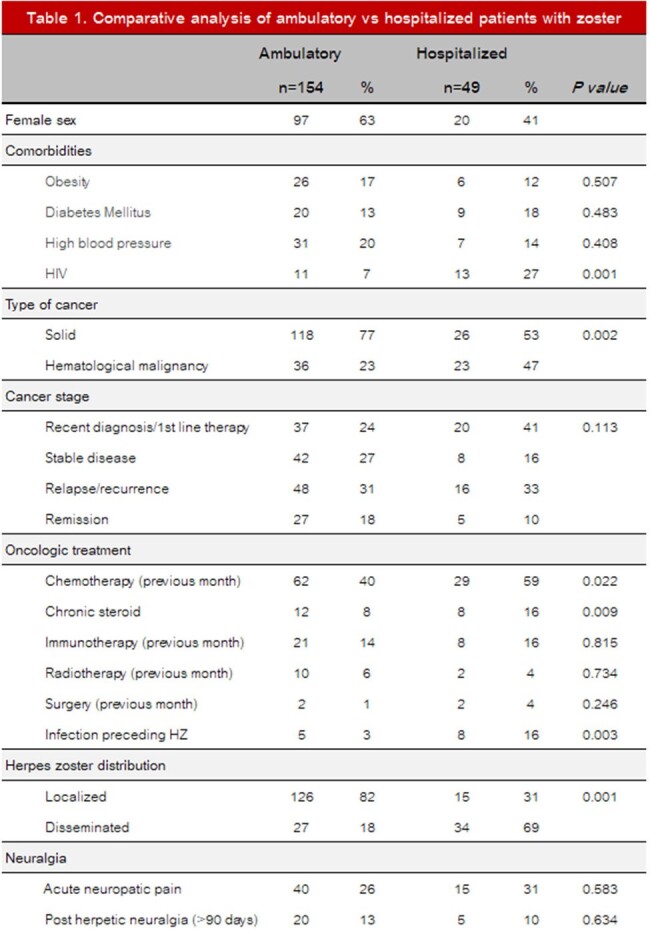

**Methods:**

Between 2021 and 2023, all patients with HZ identified at our Tertiary Cancer Institute were included. Demographics, clinical manifestations, therapeutics, and complications were registered. To determine the economic burden of the disease, an economic analysis was done.

**Figure:**
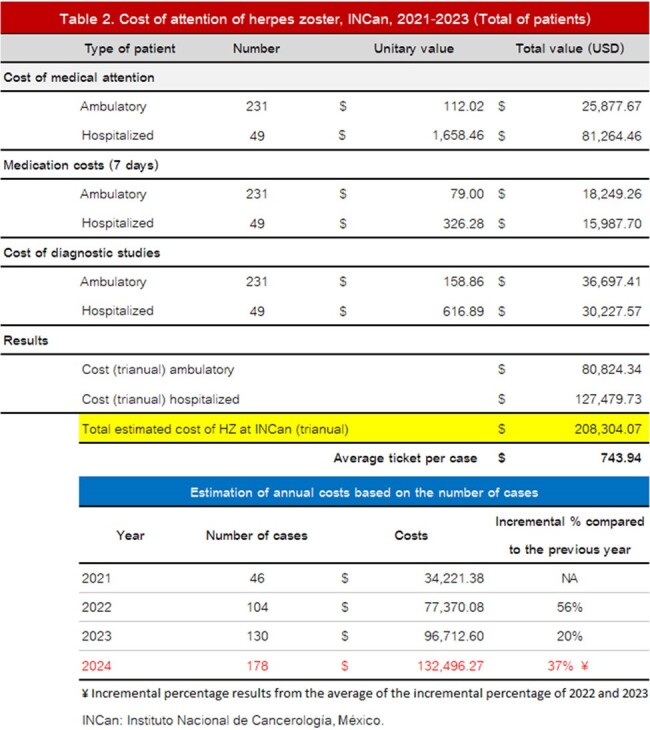

**Results:**

280 cases of HZ were identified. 164 (59%) women; median age 57 years (IQR 45-67.5). 200 solid tumors (71%) mainly breast cancer (n=72, 26%). Eighty hematological malignancies (HM) (29%), lymphoma the most frequent (n=51, 18%). 77 disseminated HZ, 62 (22%) cutaneous and 11 (4%) in CNS. 77 cases (27.5%) were treated outside of our institution. In 154 cases (55%), ambulatory therapy was prescribed, and 49 (17.5%) were treated in-hospital [median hospital stay: 8 days (IQR 5-13)]. In 45 (16%) patients, HZ delayed oncological treatment. One patient died.

Bivariate analysis comparing outpatients vs. hospitalized was significant for PLHIV (p=0.001), HM (p=0.002), chemotherapy in the previous month (p=0.022), chronic steroid (p=0.009), infection that precedes HZ (p=0.003), and disseminated zoster (p=0.001). Older age, high blood pressure, and disseminated HZ were related to the development of postherpetic neuralgia [OR 1.036 (1.006-1.066), 3.637 (1.541-8.580) and 2.961 (1.306-6.717)].

The estimated cost of care during the three years was USD 208,304.05. The average cost of care was USD 743.94. The bivariate analysis is presented in Table 1. Healthcare cost of attention is presented in Table 2.

**Conclusion:**

HZ in this oncologic center is a growing concern. The projection of the potential increase in cases for 2024 estimates 173 new cases, which will represent USD 128,419.45.

The most affected groups were patients with breast cancer, hematological malignancies, chronic steroid use, PLHIV, and those over 50 years of age. Our study's data will help enforce HZ vaccination in oncological patients and PLHIV.

**Disclosures:**

All Authors: No reported disclosures

